# Effects of the Delta Opioid Receptor Agonist DADLE in a Novel Hypoxia-Reoxygenation Model on Human and Rat-Engineered Heart Tissue: A Pilot Study

**DOI:** 10.3390/biom10091309

**Published:** 2020-09-11

**Authors:** Sandra Funcke, Tessa R. Werner, Marc Hein, Bärbel M. Ulmer, Arne Hansen, Thomas Eschenhagen, Marc N. Hirt

**Affiliations:** 1 Department of Anaesthesiology, University Medical Centre Hamburg-Eppendorf, 20246 Hamburg, Germany; s.funcke@uke.de (S.F.); t.werner@imb.uq.edu.au (T.R.W.); b.ulmer@uke.de (B.M.U.); ar.hansen@uke.de (A.H.); t.eschenhagen@uke.de (T.E.); 2 Institute of Experimental Pharmacology and Toxicology, University Medical Centre Hamburg-Eppendorf, 20246 Hamburg, Germany; 3 DZHK (German Centre for Cardiovascular Research), Partner Site Hamburg/Kiel/Lübeck, 20246 Hamburg, Germany; 4 Institute for Molecular Biosciences, The University of Queensland, Brisbane QLD 4072, Australia; 5 Anaesthesiology Clinic, RWTH Aachen University, 52074 Aachen, Germany; mhein@ukaachen.de

**Keywords:** 3D tissue model, opioids, preconditioning, translational medicine, cardioprotection, cardiac hypertrophy, reperfusion injury, human induced pluripotent stem cells, tissue engineering

## Abstract

Intermittent hypoxia and various pharmacological compounds protect the heart from ischemia reperfusion injury in experimental approaches, but the translation into clinical trials has largely failed. One reason may lie in species differences and the lack of suitable human in vitro models to test for ischemia/reperfusion. We aimed to develop a novel hypoxia-reoxygenation model based on three-dimensional, spontaneously beating and work performing engineered heart tissue (EHT) from rat and human cardiomyocytes. Contractile force, the most important cardiac performance parameter, served as an integrated outcome measure. EHTs from neonatal rat cardiomyocytes were subjected to 90 min of hypoxia which led to cardiomyocyte apoptosis as revealed by caspase 3-staining, increased troponin I release (time control vs. 24 h after hypoxia: cTnI 2.7 vs. 6.3 ng/mL, ** *p* = 0.002) and decreased contractile force (64 ± 6% of baseline) in the long-term follow-up. The detrimental effects were attenuated by preceding the long-term hypoxia with three cycles of 10 min hypoxia (i.e., hypoxic preconditioning). Similarly, [d-Ala2, d-Leu5]-enkephalin (DADLE) reduced the effect of hypoxia on force (recovery to 78 ± 5% of baseline with DADLE preconditioning vs. 57 ± 5% without, *p* = 0.012), apoptosis and cardiomyocyte stress. Human EHTs presented a comparable hypoxia-induced reduction in force (55 ± 5% of baseline), but DADLE failed to precondition them, likely due to the absence of δ-opioid receptors. In summary, this hypoxia-reoxygenation in vitro model displays cellular damage and the decline of contractile function after hypoxia allows the investigation of preconditioning strategies and will therefore help us to understand the discrepancy between successful conditioning in vitro experiments and its failure in clinical trials.

## 1. Introduction

Prolonged ischemia of the heart leads to myocardial infarction. As the duration of ischemic interval correlates with infarct size, the treatment goal is timely reperfusion with percutaneous coronary intervention. Surprisingly, additional brief episodes of ischemia before a longer ischemia interval reduce the ultimate infarct size [1], a phenomenon called ischemic preconditioning (IPC). IPC can be applied either shortly (≤2 h) or longer (24–48 h, delayed IPC) before the deleterious ischemia [2], and ischemic conditioning is also effective at the early reperfusion stage (ischemic postconditioning, IPost) [3]. Additionally, this proved that not only the ischemic phase but also the reperfusion phase contributes to the myocardial damage [4]. While IPost can be performed in the catheter lab, though posing a risk for coronary microembolization [5], ischemic conditioning has also been reported to be effective by brief occlusions of other coronary arteries or even remote organs (remote ischemic conditioning) [6]. Yet, the sufficiently powered prospective randomized controlled trial CONDI-2/ERIC-PPCI recently failed to demonstrate any benefit of remote ischemic conditioning [7].

Many ischemia-activated cardioprotective signal transduction pathways have been deciphered [2]. Based on these findings the concept evolved to activate these pathways pharmacologically. This has been shown in preclinical models for a variety of neurotransmitters, hormones, or small molecular substances, amongst them adenosine, bradykinin, natriuretic peptides, prostaglandins, and opioids [2,8]. Opioids are of particular interest as they are endogenously produced by cardiomyocytes and other cardiac cell types but are also exogenously administrable as approved drugs. Furthermore, their receptor subtypes (µ, δ, and κ) are well classified and specific agonists/antagonists exist. The δ-opioid receptor (DOR) seems most important for preconditioning, which is also underpinned by the fact that DOR-agonists can induce (incomplete) hibernation in many species, a state characterized by low cardiac energy consumption and high cardiac stress resilience [9].

While the molecular pathways of preconditioning are increasingly understood and experimental data are often encouraging, the translation into patient treatment of cardioprotection has been largely disappointing [7,10]. Supposedly, one of the reasons is that standard in vitro, but also in vivo, models are often not predictive for the clinical situation: e.g., cultured cardiomyoblasts or immortalized cells vs. primary cardiomyocytes; two-dimensional (2D) monolayer cell culture with uniform oxygen supply vs. a complex multi-billion 3D organ with uneven oxygen supply; only cardiomyocytes vs. the human heart that constitutes only 30% cardiomyocytes (by number), but also of endothelial cells, smooth muscles cells, fibroblasts, neurons, immune cells, etc. [11]; not work-performing cardiomyocytes vs. constantly loaded cardiomyocytes; rodent vs. human cells; rodent heart rates of ~300–600 bpm vs. human heart rates of ~80 bpm. Therefore, demonstrating preconditioning effects in vitro remains challenging and predictive translational models are rare.

In this article, we present a novel in vitro model that tackles some of the aforementioned shortcomings. A 3D cell culture format of primary rat cardiac cells (multi-cell types) called engineered heart tissue (EHT), which constantly beats and performs work, was exposed to hypoxia and reoxygenation. While most former studies stopped a few hours after reperfusion, we extended the follow-up period to many days. Finally, we humanized the model by repeating key experiments with EHTs from human induced pluripotent stem cell (hiPSC)-derived cardiomyocytes.

## 2. Materials and Methods

### 2.1. Generation of Rat and Human Engineered Heart Tissue (rEHT/hEHT)

The procedures to generate rat EHT have been described in detail previously [12,13]. They have been reviewed and approved by the regional ethics review board of the Medical Council of Hamburg, Germany (approval number ORG516), and were conducted in accordance with the Guide for the Care and Use of Laboratory Animals as adopted by the United States National Institutes of Health (8th edition, revised 2011) and with the ARRIVE (Animal Research: Reporting of In Vivo Experiments) guidelines. In contrast to rat EHTs containing all types of heart cells, human EHTs were generated only of cardiomyocytes (with a small percentage of non-myocytes) derived from hiPSCs (approved by the regional ethics review board of the Medical Council of Hamburg, Germany, approval number PV4798, all donors gave written informed consent) by reprogramming human fibroblasts [14]. A summary of these procedures can be found in the Supplementary Materials (Method S1).

The final result of these procedures is EHTs, consisting of heart cells embedded in a fibrin matrix, spanned between two flexible silicone posts. Until the day of hypoxia-reoxygenation experiments EHTs were cultured in 1.5 mL of baseline low-glucose medium. A detailed description of different culture media is provided in the Supplementary Materials (Method S2). After each medium change (three times a week), the spontaneous contractile force of EHTs was determined in a semi-automated contractility measurement system (Supplementary Materials, Figure S1). A prerequisite for the hypoxia-reoxygenation experiments was that contractile forces had reached a plateau, which usually occurred around day 20–25.

A list with detailed information on sources of compounds, reagents, cell lines and equipment used is provided in the Supplementary Materials (Table S1).

### 2.2. Induction of Hypoxia-Reoxygenation Injury and Hypertrophy

**Figure 1:**
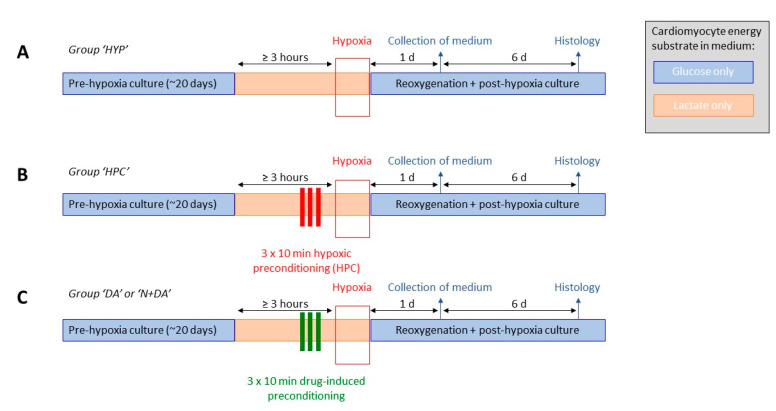
Study design and different hypoxia-reoxygenation experimental groups. (**A**) Hypoxia-reoxygenation alone (HYP) or (**B**) preceded by hypoxic preconditioning (HPC) or (**C**) preceded by drug-induced preconditioning (with [d-Ala2, d-Leu5]-Enkephalin (DADLE): DA, or Naloxone and DADLE: N+DA).

Figure 1 gives an overview of the study protocol and experimental groups. Three hours before the onset of the long-term hypoxia (Figure 1A) or the brief hypoxic preconditioning cycles (Figure 1B) the EHTs were transferred from baseline low glucose culture medium to lactate medium (Supplementary Materials, Method S2) with 4 mM lactate as a single energy source. For hypoxic preconditioning (Figure 1B, group “HPC”), culture dishes filled with lactate medium were put into a hypoxia cell culture incubator (5% O_2_) for ≥ 3 h for full gas equilibration. EHTs were transferred into these culture dishes in the hypoxia incubator for 10 min and then back into non-hypoxic cell culture dishes for 10 min. This procedure was repeated for 3 cycles. After hypoxia, EHTs were transferred to high oxygen per equilibrated serum-free low-glucose DMEM (Supplementary Materials, Method S2).

Accordingly, for drug-induced preconditioning (Figure 1C), EHTs were transferred into culture dishes containing lactate medium supplemented with 100 nM of the δ-opioid receptor agonist [d-Ala2, d-Leu5]-enkephalin (DADLE; group “DA”). Additionally, the nonspecific opioid receptor antagonist naloxone (10 µM) was added to DADLE in a second group (group “N+DA”). EHTs remained in the medium for 10 min and were then transferred back into drug-free cell culture dishes for 10 min. These steps were also repeated in 3 cycles.

The long-term hypoxia and reoxygenation to induce tissue injury were performed inside the automated contractility measurement system (Figure S1). One of its components is a small incubator (volume 15.3 L) with a controllable gas inflow and a passive gas outflow. In the hypoxic periods, the gas composition was 93% N_2_ and 7% CO_2_.

### 2.3. Mechanical Pro-Hypertrophic Intervention in EHTs

A pathological type of cardiac hypertrophy, including contractile impairment and adverse remodeling, can be evoked by a prolonged sharp increase in the afterload of EHT [13]. Before the intervention, the serum content of the medium was decreased gradually from 10% over 4% (for two days) to 0%, (Supplementary Materials, Method S2). The afterload enhancement by a factor of 12 was induced by stiffening the flexible hollow silicone posts, which serve as the suspension of EHTs, by very rigid metal braces and was performed for one week. A detailed protocol of this hypertrophic intervention has been published previously [13].

### 2.4. Quantification of Cellular Damage

Cardiac tissue injury was measured by quantifying the release of cardiac troponin I into the culture medium by a high-sensitivity rat cardiac Troponin I ELISA Kit (Life Diagnostics Inc., CTNI-2-HSP). Furthermore, cell death was determined by measuring the release of the cytosolic enzyme glucose-6-phosphate-dehydrogenase (G6PDH) from damaged cells into the surrounding culture medium with the Vibrant Cytotoxicity Assay Kit (Molecular Probes Europe BV, V-23111). The release of H_2_O_2_ into the cell culture medium was quantified by the ROS-GLO H_2_O_2_ Assay (Promega, G8820). All kits were carried out according to the manufacturer’s recommendations and analyzed with either a microplate reader (TECAN Safire II, Tecan Trading) or a microplate luminometer (Centro LB 960, Berthold Technologies).

### 2.5. Immunohistochemistry

EHTs were fixed for 24 h in a phosphate-buffered solution containing 4% formaldehyde stabilized with methanol. After embedding in paraffin, 3 µm sections were cut longitudinally in the median plane. Staining conditions for each antibody had been established on rat heart tissue samples. The conditions for rat EHTs were: mouse anti myosin light chain 2, ventricular isoform (MLC-2V) monoclonal antibody (Synaptic Systems, 310111), dilution 1:2000, antigen retrieval: 30 min in citrate buffer, pH 6.0; mouse anti-ANP (atrial natriuretic peptide) (23/1) monoclonal antibody (Santa Cruz, sc-80686), dilution 1:500, antigen retrieval: 60 min in citrate buffer, pH 6.0; rabbit anti-active caspase-3 polyclonal antibody (R&D Systems, AF835), dilution 1:300, antigen retrieval: 60 min in CC1-solution (Roche, 950-124). All antibodies were visualized with the multimer-technology based UltraView Universal DAB Detection Kit (Roche, 760-500). All microscopic images were taken on an Axioskop 2 microscope (Carl Zeiss Microscopy GmbH). The quantification of images was performed in ImageJ. ANP positive cells were counted with the “magic tool” at an 8-bit b/w threshold of 190. For the quantification of active caspase-3 positive (dark brown) nuclei, the settings were: 8-bit b/w threshold 120, analyzed particle size 30–120 µm^2^, analyzed particle circularity 0.3–1.0.

### 2.6. Isolation of RNA and qPCR from EHTs

EHTs or human cardiac tissue samples (reviewed and approved by the regional ethics review board of the Medical Council of Hamburg, Germany, approval number 088/04) were homogenized and total RNA was extracted. For reverse transcription of mRNA and the subsequent PCR a cDNA reverse transcription kit was used according to the manufacturers’ instructions. PCR products were visualized on a 2%-agarose gel with DNA stain (for more details see Supplementary Materials, Method S3).

### 2.7. Statistical Analysis

This is an exploratory pilot study. Consequently, the sample size was based on the authors’ previous experience with the EHT model being used. Thus, no statistical power calculation was conducted before the study. Group sizes varied (*n* = 6 to 12 per group) depending on how many groups were aimed to be compared, as the total sample size of each experiment was limited to 24 due to the usage of 24-well plates in the contractility measurement system. Furthermore, the 3D cell constructs are sensitive to mechanical stress (e.g., transfer at media changes) and in case of loss of EHTs they were excluded from the final analysis. Rupture of the EHTs and errors in semi-automatic force recording were the only causes for missing data. For some of the further analyses (e.g., troponin ELISA) data were pooled from EHTs from the respective treatment groups to facilitate analysis in replicates to increase test validity.

Results are presented as mean ± SEM. All statistical tests were performed in GraphPad Prism version 6.01. or 8.20 (GraphPad Software). In detail, 1-way ANOVA and Dunnett‘s multiple comparison post-test (to compare to controls) were used for more than 2 groups or Student’s unpaired t-test for 2 groups. When two factors affected the result (e.g., time and preconditioning), 2-way ANOVA analyses and Sidak’s multiple comparisons test were performed. *p* < 0.05 or less was considered statistically significant. *P*-values are displayed graphically as follows: * *p* < 0.05, ** *p* < 0.01, *** *p* < 0.001, ns = not significant.

## 3. Results

### 3.1. Optimization of the Experimental Procedure to Obtain a Reliable Hypoxic Trauma in EHTs

**Figure 2:**
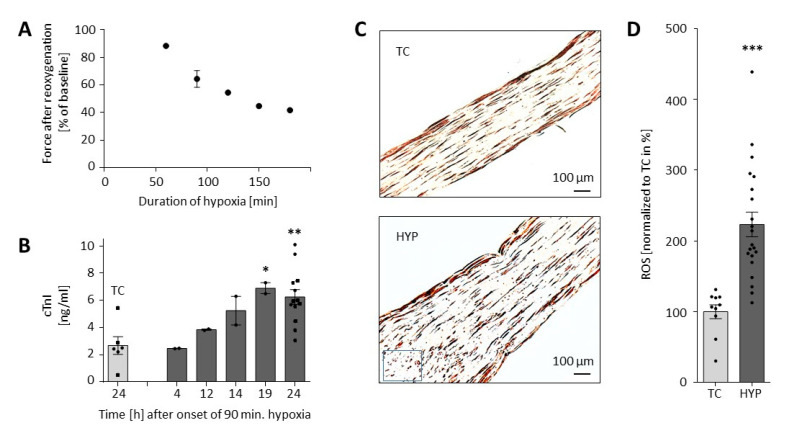
Model of hypoxia-reoxygenation injury in rat-engineered heart tissues (EHTs). (**A**) Correlation between duration of hypoxia and decrease in force after 120 min of reoxygenation (each dot represents the mean force of one batch of EHTs (*n* = 8–12), except for 90 min where hypoxia experiments from 9 independent batches were averaged (mean ± SEM). (**B**) Release of cardiac troponin I (cTnI) over 24 h after the onset of hypoxia (90 min) compared to the time control (TC) group. Data are presented as scatterplot with mean (bars) ± SEM (whiskers). One-way ANOVA with Dunnett‘s post-hoc test (*n* = 2–13, each measurement from the pooled cell culture media of 2 EHTs; “19 h” vs. “TC”, * *p* = 0.029; “24 h” vs. “TC”, ** *p* = 0.002). The values depicted as circles stem from one experimental series. (**C**) MLC-2V (myosin light chain 2, ventricular isoform) staining of EHTs undergoing 90 min of hypoxia and reoxygenation (HYP) and control group without hypoxia (time control = TC). In some central areas of the hypoxic EHTs (e.g., at the blue rectangle) only roundish cardiomyocytes were detectable. (**D**) Reactive oxygen species (ROS) measured in the cell culture media of TC and HYP groups. Student’s unpaired t-test (*n* = 10–21 per group, *** *p* < 0.0001).

In pilot experiments, EHTs were successfully cultivated with lactate instead of glucose as the single energy substrate for more than one week without noticeable changes in contractile behavior. There was an inverse relationship between the duration of hypoxia and the forces in the plateau phase 120 min after the initiation of reoxygenation (Figure 2A). All 90 min hypoxia experiments together showed a recovery of the contractile force to 64 ± 6% (*n* = 9 independent batches of 8–12 EHTs/batch) of baseline. For all further experiments, we chose a duration of 90 min. Cardiac damage was also shown by a continuous increase in cumulative cardiac troponin I in the culture medium from hour 4 to 24 after the onset of reoxygenation (Figure 2B). Following another week of culture, cardiomyocytes in EHTs were visualized by MLC-2V staining. Compared to time controls, EHTs that underwent 90 min of hypoxia revealed lower densities of elongated cardiomyocytes and more necrotic or apoptotic (i.e., roundish) cardiomyocytes (Figure 2C) and released more reactive oxygen species (ROS) into the cell culture medium (Figure 2D).

### 3.2. Preconditioning of EHTs by Brief Episodes of Hypoxia

**Figure 3:**
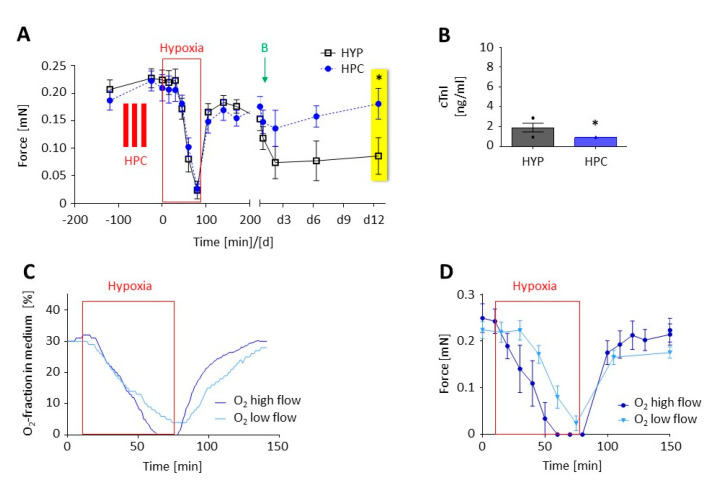
(**A**,**B**) Effect of hypoxic preconditioning (HPC, 3 × 10 min) followed by 90 min hypoxia compared to the hypoxic group without preconditioning (HYP). Data are presented as mean ± SEM. (**A**) Force (mN) before, during and after hypoxia in early (0–2 h) and late (≥2 days) reoxygenation. Two-way ANOVA and Sidak’s post-test at day 12 (yellow box, *n* = 10 per group, * *p* = 0.024). (**B**) Release of cardiac troponin I (cTnI) in the first 24 h (see arrow in A) after reoxygenation. Unpaired t-test (*n* = 6–7, * *p* = 0.031). (**C**,**D**) Impact of gas flow rates on the O_2_-fraction in the medium and on force. (**C**) O_2_-fraction measured in the medium during hypoxia experiments at high (5 L/min) or low gas flow (0.5 L/min) in the hypoxic chamber. The oxygen concentration inside the incubator (gas phases), as well as in the medium (5 mm below the surface, liquid phase), was determined with an oxygen microprobe based on fluorescent light emission (Microx-TX transmitter, tip diameter < 50 µm; Presens Precision Sensing GmbH, Regensburg, Germany). (**D**) Corresponding changes of the contractile force over time.

We exposed rat EHTs to 90 min of hypoxia in lactate medium. In the treatment group, the EHTs underwent three cycles of 10 min short hypoxic preconditioning (group “HPC”). Figure 3A shows that contractile force in both groups decreased during hypoxia and increased during early reoxygenation (0–2 h) to a similar extent. In the follow-up period (late reoxygenation, ≥2 days) the force was preserved only in the group of the preconditioned EHTs. Cardiac troponin I levels in the medium were significantly lower in the HPC group (Figure 3B). We measured the oxygen fraction of the gas dissolved in the medium depending on the input gas flow into the contractility measurement system. The oxygen fraction in the medium (Figure 3C) was highly dependent on the inflow rate and considerably lagged behind the O_2_-fraction in the air. The forces of the EHTs (Figure 3D) reflected this tardy decrease in the O_2_-fraction in the medium under the low-flow more than under the high-flow rate. We thus used a consistent protocol in all following experiments with a high-flow rate for the first 10 min of hypoxia to ensure the fast decrease in the O_2_-fraction in the medium and then returned to the low-flow rate to maintain the oxygen mixture.

### 3.3. Preconditioning with a δ-opioid Receptor Agonist (DADLE)

**Figure 4:**
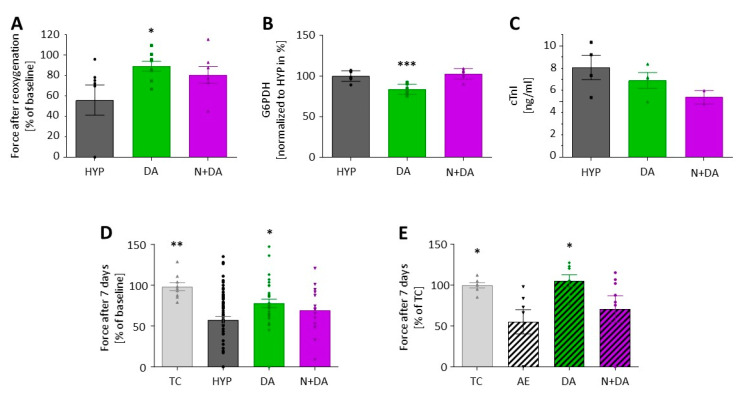
(**A**–**D**) Effect of preconditioning with [d-Ala2, d-Leu5]-Enkephalin (DADLE) 100 nM, 3 × 10 min, (DA) or DADLE 100 nM plus naloxone 10 µM (N+DA) compared to the hypoxic group without preconditioning (HYP) and the time control (TC) without hypoxia. Data are presented as scatterplot with mean (bars) ± SEM (whiskers). One-way ANOVA with Dunnett‘s multiple comparisons test, in panels A–D compared to HYP group. (**A**) Force in early reoxygenation (120 min after the onset of reoxygenation) as percent of initial force before hypoxia (*n* = 7–8, “DA” vs. “HYP” * *p* = 0.046; “N+DA” vs. “HYP”, *p* = 0.165). (**B**) Hypoxia-reoxygenation injury (of experiment depicted in (**A**)) quantified by glucose-6-phosphate dehydrogenase (G6PDH) release in the first 24 h after onset of hypoxia normalized to the amount of G6PDH in the hypoxia group without preconditioning (*n* = 5; “DA” vs. “HYP”, *** *p* = 0.0003). (**C**) Release of cardiac troponin I (cTnI) in the first 24 h (of experiment depicted in A) after reoxygenation, *n* = 3 samples per group, each sample pooled from media from 2 EHTs. (**D**) Force in late (7 days) reoxygenation as percent of initial force before hypoxia (TC *n* = 9, HYP *n* = 71, DA *n* = 37, N+DA *n* = 15; “TC” vs. “HYP”, ** *p* = 0.003; “DA” vs. “HYP”, * *p* = 0.012; “N+DA” vs. “HYP”, *p* = 0.523). (**E**) Reduction in force (relative force in % of baseline compared to the time control) after 7 days of afterload enhancement (hatched bars) without (AE) and with preconditioning (DA and N+DA, *n* = 7–8 per group; “TC” vs. “AE”, * *p* = 0.044; “DA” vs. “AE”, * *p* = 0.022; “N+DA” vs. “AE”, *p* = 0.682; 1-way ANOVA with Dunnett‘s multiple comparisons test compared to group AE).

To explore drug-induced preconditioning, we used the δ-opioid receptor agonist [d-Ala2, d-Leu5]-enkephalin (DADLE). Again, we exposed rat EHTs to 90 min of hypoxia in lactate medium and reoxygenation in glucose medium (group “HYP”). This time the EHTs in the treatment group were incubated in lactate medium containing 100 nM DADLE (group “DA”) or 100 nM DADLE plus 10 µM naloxone (group “N+DA”) for three cycles of 10 min preconditioning. Contractile force recovered significantly better in the DA group than in the HYP group (Figure 4A). This effect was partly antagonized by naloxone. Accordingly, in the DA group G6PDH release was lower compared to the HYP group (Figure 4B). The cTnI analyses revealed no statistically significant differences between all groups (Figure 4C). The protective effect of DADLE preconditioning was maintained. Seven days after induction of hypoxia-reoxygenation injury the force in the DA group was lower than the time control without hypoxia, but significantly better than the hypoxic HYP group without conditioning (Figure 4D).

**Figure 5:**
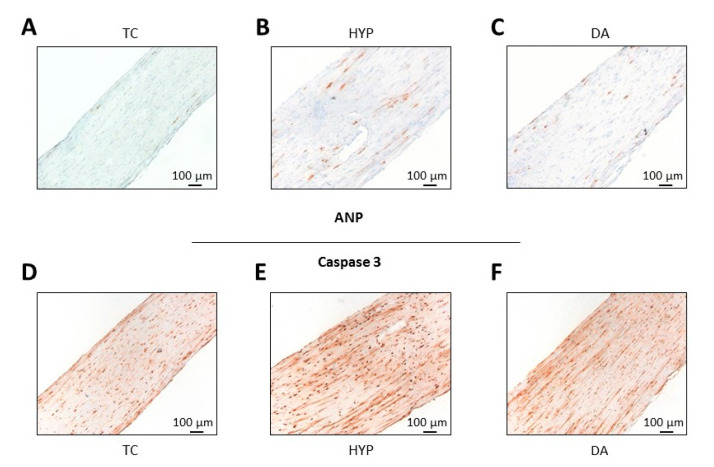
Effect of preconditioning with [d-Ala2, d-Leu5]-Enkephalin (DADLE, 100 nM, 3 × 10 min, DA), followed by 90 min hypoxia compared to the hypoxic group without preconditioning (HYP) and to the control group without hypoxia (time control, TC). (**A**–**C**) Atrial natriuretic peptide (ANP)-stained paraffin section of the EHT in a longitudinal view. (**A**) 5.0 ANP^+^ cells/mm^2^ in TC, (**B**) 31.1 ANP^+^ cells/mm^2^ in HYP, (**C**) 15.0 ANP^+^ cells/mm^2^ in DA. (**D**–**F**) Active Caspase 3 (subunit p17)-stained paraffin section of EHTs in a longitudinal view. (**D**) 37.7 Casp-3^+^ nuclei (dark brown)/mm^2^ in TC, (**E**) 222.2 Casp-3^+^ nuclei/mm^2^ in HYP, (**F**) 43.1 Casp-3^+^ nuclei/mm^2^ in DA. Nuclei in (**A**–**F**) were lightly counterstained with hematoxylin.

This was also reflected in representative micrographs of ANP- (Figure 5A–C) and Caspase 3-stained (Figure 5D–F) paraffin sections of the EHTs. Hypoxia led to an increase in ANP- and Caspase 3-positive cells accentuated in the central parts of the EHT (Figure 5B,E). The number of ANP-positive cells representing stressed cardiomyocytes and Caspase 3-positive (i.e., apoptotic) cells was lower when EHTs were preconditioned with DADLE (Figure 5C,F). To test if the protective long-term effect of DADLE also holds true for other types of cardiac stress, we performed a strong increase in afterload of EHTs [13]. DADLE fully prevented the afterload-induced decline in force. This effect was partly reversed by the addition of the opioid receptor antagonist naloxone (Figure 4E).

### 3.4. Transfer of the Hypoxia-Reoxygenation Protocol to Human EHTs

**Figure 6:**
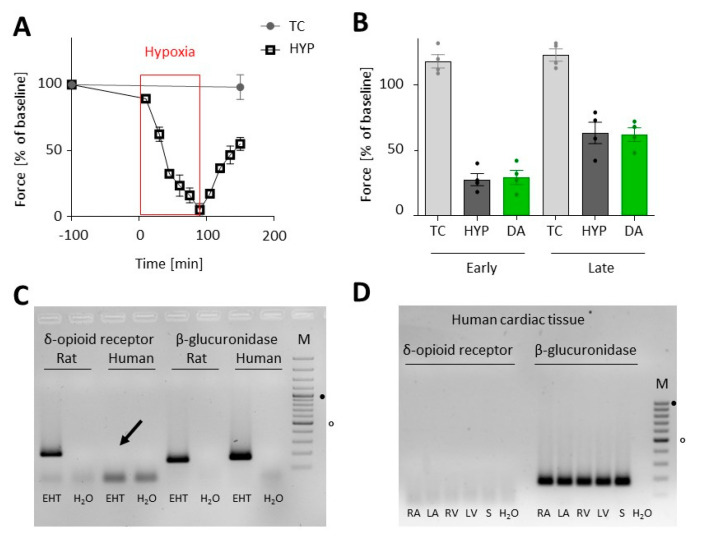
Hypoxia-reoxygenation injury in human EHTs. (**A**) 90 min of hypoxia (HYP) followed by reoxygenation in human EHTs (vs. time control without hypoxia, TC) (mean ± SEM). (**B**) Effect of preconditioning by [d-Ala2,d-Leu5]-Enkephalin (DADLE, 100 nM, 3 × 10 min, DA) followed by 90 min hypoxia compared to the hypoxic group without preconditioning (HYP) and TC on the contractile force (shown in % of the force before hypoxia at baseline) in the early (120 min after the onset of reoxygenation) and late (day 1) reoxygenation, compared by 1-way ANOVA with Sidak‘s multiple comparisons test (HYP vs. DA, *n* = 4, *p* = 0.812 (early) and *p* = 0.891 (late)). Data are presented as scatterplot with mean (bars) ± SEM (whiskers). (**C**,**D**) PCR analyses of δ-opioid receptor expression. β-glucuronidase served as positive, water as negative control. M = marker, indicates 500 bp and 1000 bp size. (**C**) Detection of the δ-opioid receptor in rat in contrast to human EHTs. The arrow indicates the lack of a δ-opioid receptor signal in human EHTs. (**D**) PCR analysis showing absence of δ-opioid receptor mRNA in human atrial (RA = right atrium, LA = left atrium), septal (S) and ventricular (RV = right ventricle, LV = left ventricle) tissue samples.

Lastly, the implemented treatment protocol was adapted from rat to human EHTs (Supplementary Materials, Video S1). We exposed human EHTs to 90 min of hypoxia in lactate medium. All culture and preconditioning steps before were identical to those described for rat EHTs. Likewise, 90 min of hypoxia and reoxygenation led to a comparable reduction in force in human, as in rat, EHTs (Figure 6A). However, in contrast to rat EHTs, no better recovery after DADLE preconditioning could be observed in neither early nor late reoxygenation (Figure 6B). PCR analysis revealed that the δ-opioid receptor was not detectable in human EHT tissue (Figure 6C), as well as in human atrial, septal, and ventricular tissue samples (Figure 6D).

## 4. Discussion

In this study, we established a model with a reproducible extent of functional deterioration and cell damage following hypoxia-reoxygenation injury in 3D EHTs. We observed a comparable amount of hypoxic trauma in rat and human EHTs with fixed duration of hypoxia. Further, hypoxic preconditioning was shown to be possible in rat EHTs. The cardioprotective delta opioid receptor agonist DADLE added to the culture medium led to an improvement of recovery of contractile force in the early reperfusion in rat EHTs. This effect was preserved until the latest analyzed time point (at 7 days after the start of reoxygenation) and it was partially antagonized by the nonselective opioid receptor antagonist naloxone. This protective long-term effect of DADLE was not limited to hypoxia-reoxygenation injury, but also effective in the attenuation of afterload enhancement-induced contractile impairment. DADLE had no protective effect in human EHTs, consistent with absence of DOR expression in hiPSC-EHTs.

Cardiac hypoxia and reoxygenation can lead to tremendous loss of cardiomyocytes and ultimately to heart failure. Preclinical models have demonstrated protective strategies to reduce the cardiac damage [15]. Though many details of the underlying protective mechanisms and complex intracellular pathways of ischemic and drug-mediated conditioning have been revealed during the past decades, the transition into the clinical setting has been mostly disappointing. Thus, there is an ongoing need for improved models that help to better predict the effects of conditioning in the human body. Three-dimensional cell culture systems better describe the in vivo behavior of most cell types than 2D cell types [16,17]. Engineered heart tissue (EHT) from neonatal rat heart cells develop a high degree of cellular differentiation, intercellular coupling, longitudinal orientation, and force generation [12,18,19]. Therefore, it may be used as a drug screening platform. In recent years, it also became possible to generate cardiac myocytes from hiPSC [20,21]. While differentiation protocols are increasingly efficient, hiPSC-cardiomyocytes in 2D remain immature. Their maturity can also be substantially ameliorated by long-term and 3D culture (i.e., the EHT format) [22]. Thereby, disease mechanisms could finally be experimentally investigated in a human context.

As even 3D models still provide a non-physiological setting in vitro, we first had to validate that hypoxia-reoxygenation injury is inducible. While our previous studies failed to produce persistent cellular damage under hypoxic conditions in glucose medium [23], we were able to simulate a state of intermittent ischemia in vivo more closely by changing from glucose to lactate as energy metabolite in the culture medium. Notably, 90 min of hypoxia in the air phase sufficed to induce long-term cellular damage and functional impairment, which is similar to the known time frame in animal studies [24] and much shorter than that used in other studies [23,25,26].

Though hypoxic preconditioning was not the primary scope of our investigations it is still the best investigated protective stimulus. We observed that hypoxic preconditioning in rat EHTs preserved force in the follow-up period (late reoxygenation; Figure 3A), but not immediately after reoxygenation. Functional superiority in the follow-up period was associated with lower troponin levels measured after the first 24 h (Figure 3B). This is in line with in vivo studies with a reduction in infarct size by hypoxic preconditioning [1].

The role of opioids in protection from ischemia-reperfusion injury became visible when IPC was found to be inhibited by opioid receptor antagonists [27] and mimicked by opioids [28]. From the major three single-gene derived opioid classes only delta, barely kappa and almost no mu-subtypes are expressed in the rat heart [29]. Therefore, we investigated the DOR agonist [d-Ala2, d-Leu5]-enkephalin (DADLE). In our study, DADLE positively influenced the recovery of contractile force of the rat EHTs, both in the early and in the late reperfusion period (Figure 4A,D). Consistent with the functional improvement, G6PD levels were lower in the DADLE group indicating less acute necrotic cell death (Figure 4B). ANP- and Caspase 3-staining revealed that cardiomyocytes located in the center of the EHT were more at risk in case of hypoxia (Figure 5). This central fading of cardiomyocytes occurred less after pre-treatment with DADLE.

The positive effects of DADLE preconditioning were antagonized by the nonselective opioid receptor blocker naloxone (Figure 4), but the effect was incomplete. One possible explanation could be the relatively high expression of the naloxone-metabolizing enzyme UGT2B7 in rat hearts (~10% of the liver expression) [30]. Another explanation might be the 65-fold lower affinity of naloxone to DOR than to µ-opioid receptors [31]. In both cases, one would expect a more complete effect by an even higher concentration of naloxone (which would increase the risk of off-target or toxic effects) or better by employing a more specific antagonist of DOR, such as naltrindole.

Recently, Chen and Vunjak-Novakovic [25] published a model that fits well with ours. While this group focused on cell or mitochondrial viability markers (LDH, AK, RealTime Glo-assay and JC1) as readout for protective strategies, we employed different markers (troponin I, G6PDH and ROS), but foremost we emphasized contraction analyses and extended the follow-up period to a maximum of 12 days. The latter seems important because other conditioning agents such as xenon improved cardiac remodeling and contractile function after ischemia-reperfusion only in long-term functional measurements [32]. To follow the hypothesis on delayed effects on cardiac remodeling, we tested DADLE in a mechanical pro-hypertrophic intervention (afterload enhancement) and discovered similar protective effects (Figure 4E). This underpins the versatility of the EHT system to investigate cardioprotective substances not only for the prevention of hypoxia-reoxygenation injury, but also for the prevention of adverse remodeling which we have previously shown, e.g., for endothelin receptor antagonists [13], micro-RNA-antagonists [33,34] or TGFβ-inhibitors [35].

The transition of the hypoxia-reoxygenation injury model from rat cardiac cells to human cardiomyocytes differentiated from iPSCs was straightforward and without modifications to previously published protocols [14,36]. Again, 90 min of hypoxia in the air phase led to halving of contractile forces in the long term, a phenomenon which we have also observed in another project [37]. DADLE has been shown to have cardioprotective properties in isolated human right atrial trabeculae [38,39]. Nevertheless, there are controversial data about the distribution and protein abundance of DOR in human heart. Some studies detected DOR protein in cardiac and/or neuronal cells [40,41]. In one of the studies a DOR agonist had no protective action in the absence of ICA cell-ventricular myocyte co-culture during ischemia-reperfusion [40]. Yet, DADLE did not protect from hypoxia-reoxygenation injury in human EHTs other than in rat EHTs that might simply be explained by the absence of DOR in human EHTs. This notion is supported by RNAseq-data reporting moderate expression of DOR in rat hearts [30], but neither detectable DOR expression [42] nor protein abundance in human hearts (www.proteinatlas.org) [43]. This is in line with our own PCR findings from human cardiac tissue samples from various locations (both atria, ventricles and septal wall) of the human heart. In contrast, Lendeckel et al. found atrial mRNA expression of opioid peptide precursors and receptors in human atrial tissue [44]. Thus, it could be of interest to test if recently described atrial-like human EHTs [45] express the DOR and react to DADLE.

Another prominent example for species differences is adenosine signaling which is important in preconditioning in rabbits [46], sheep [47] and human trabeculae [48] but not in rats [49]. The different findings in rat vs. human EHTs highlight species differences, which may account for some of the problems in translating preclinical findings of cardioprotection to humans. In addition, rat and human EHTs might help in elucidating which opioid receptors confer cardioprotection in the respective species, as there is currently a multitude of conflicting data [27,50].

There clearly are some limitations that need to be addressed. As we were looking for treatment options that would be implementable in the clinical routine, we have not tested hypoxic preconditioning in human EHTs yet. In addition, more cardioprotective approaches need to be tested, especially pharmacological postconditioning. Another current limitation of our human EHTs is their consistence of only cardiomyocytes (and in our experiments about 15% of non-myocytes), that could be solved by the supplementation with other cardiac cell types. Obviously, even rat EHTs with all cardiac cell types are still lacking in fully mirroring in vivo physiology, by e.g., absence of microvascular effects and no contribution from blood components such as platelets and immune cells.

## 5. Conclusions

Taken together, we present a new model to compare hypoxia-reoxygenation injury in rat and human EHTs. The amount of injury is quantifiable by troponin or G6PDH release and observable with histological stainings, but most importantly by changes in contractility, which can be monitored before and during hypoxia, as well as during and after reoxygenation at any time point and as frequently as desired. We and others in a similar model [24] have shown that hypoxic or drug-induced preconditioning strategies are effective in rat EHTs. Our model is an attempt to reduce the translational gap by using a three-dimensional culture format instead of conventional monolayer cultures and by the exciting possibility to use human instead of rodent cells to investigate conditioning phenomena.

## Supplementary Materials

The following are available online at https://www.mdpi.com/2218-273X/10/9/1309/s1, Method S1: Generation of rat and human EHT; Method S2: Detailed information on the different culture media; Method S3: Isolation of RNA and qPCR from EHTs. Figure S1: Contractility measurement system. Table S1: Sources of compounds, reagents, cell lines and equipment used. Video S1: Video of a spontaneously beating human EHT.
