# Supplementary material for: Effects of the Delta Opioid Receptor Agonist DADLE in a Novel Hypoxia-Reoxygenation Model on Human and Rat-Engineered Heart Tissue: A Pilot Study
Source: Biomolecules. 2020 Sep 11;10(9):1309. doi: 10.3390/biom10091309 (PMC7565486; doi:10.3390/biom10091309)
Supplement: Supplementary file 1 [file biomolecules-10-01309-s001.zip › biomolecules-897942-supplementary materials/Supplementary materials.pdf]

Supplementary Material

Effects of the Delta Opioid Receptor Agonist DADLE in a Novel Hypoxia-Reoxygenation Model on Human and Rat-Engineered Heart Tissue: A Pilot Study

Sandra Funcke, Tessa R. Werner, Marc Hein, Bärbel M. Ulmer, Arne Hansen, Thomas Eschenhagen and Marc N. Hirt

Method S1: Generation of rat and human EHT

In short, neonatal rats (postnatal day 0 to 3) were rapidly decapitated, hearts were excised and ventricular rat heart cells (all cell types, not only cardiomyocytes) were isolated by a Trypsin/DNase digestion. 500,000 cells per EHT were mixed with fibrinogen and thrombin at a final volume of 100 µl and then quickly pipetted into agarose casting moulds, into which two hollow silicone posts protruded. After 1-2 hours, the fibrin block containing the heart cells could be taken out of the moulds with the help of the two silicone posts, in between which the fibrin strip was spanned. Subsequently, these - now called - EHTs were transferred to 24-well culture dishes filled with 1.5 ml medium per well. Until the day of hypoxia-reoxygenation experiments EHTs were cultured in medium consisting of low glucose (1 g/L) Dulbecco's Modified Eagle's - Medium (DMEM, Biochrom F0415), 10% inactivated horse serum (Gibco 26050), penicillin/streptomycin (each 100 U/mL, Gibco 15140), insulin (10 µg/mL, Sigma-Aldrich 857653) and aprotinin (33 µg/mL, Sigma-Aldrich A1153, to prevent rapid fibrin degradation). Both the contractility measurement system and the cell culture incubators were set at 90% humidity, 37 °C, 7% CO_2 and 40% O_2 before and after the hypoxic periods.

For hiPSC experiments an in-house control cell line derived from a healthy female donors was utilized. Cell line authentication is UKEi003-C. Microbiological sterility, genetic stability, pluripotency and potency was verified, information accessible at hpsreg.eu.

After expansion, hiPSCs were differentiated into human cardiomyocytes in a multi-step-procedure (embryoid body formation at day 0, mesoderm induction at day 1, cardiac differentiation starting from day 4), all in suspension culture (Breckwoldt K, Letuffe-Breniere D, Mannhardt I, et. al.: Differentiation of cardiomyocytes and generation of human engineered heart tissue. *Nat Protoc* 2017; 12: 1177-1197). Between day 14 and 17, the embryoid bodies were dissociated into cardiomyocytes by collagenase II. Apart from using double the amount of cells per EHT (1,000,000 human cardiomyocytes) and the addition of matrigel (10% final volume of an EHT, BD Bioscience 356235) all casting and culture steps were identical to those described above in the rat EHT section. Flow cytometry analysis revealed a content of 83- 85% cTNT positive cells.

Method S2: Detailed information on the different culture media

<u>Baseline low-glucose medium</u>: low glucose (1 g/L) DMEM (Biochrom F0415), 10% inactivated horse serum (Gibco 26050), penicillin/streptomycin (each 100 U/mL, Gibco 15140), insulin (10 µg/mL, Sigma-Aldrich 857653) and aprotinin (33 µg/mL, Sigma-Aldrich A1153, to prevent rapid fibrin degradation); relating to main manuscript methods section on *"Generation of rat and human engineered heart tissue (rEHT/hEHT)"*.

<u>Lactate medium during hypoxia:</u> lactate (Caesar and Loretz GmbH, 7455) at 4 mM was added to minimal DMEM (A14430-01, Gibco), penicillin/streptomycin (each 100 U/mL, Gibco 15140), insulin (10 µg/mL, Sigma-Aldrich 857653), aprotinin (33 µg/mL, Sigma-Aldrich A1153), triiodothyronine 0.5 ng/mL and hydrocortisone 50 ng/mL; for treatment purposes eventually supplemented with 100 nM of the δ-opioid receptor agonist [D-Ala^2, D-Leu^5]-enkephalin and/or the nonspecific opioid receptor antagonist naloxone (10 µM), relating to main manuscript methods section on *"Induction of hypoxia-reoxygenation injury and hypertrophy"*.

<u>Reoxygenation low-glucose medium:</u> low glucose (1 g/L) DMEM (Sigma-Aldrich F0415), penicillin/streptomycin (each 100 U/mL, Gibco 15140), insulin (10 µg/mL, Sigma-Aldrich 857653), aprotinin (33 µg/mL, Sigma-Aldrich A1153), triiodothyronine 0.5 ng/mL and hydrocortisone 50 ng/mL; relating to main manuscript methods section on *"Induction of hypoxia-reoxygenation injury and hypertrophy"*.

<u>Afterload enhancement medium:</u> low glucose (1 g/L) DMEM (Sigma-Aldrich F0415), penicillin/streptomycin (each 100 U/mL, Gibco 15140), insulin (10 µg/mL, Sigma-Aldrich 857653) and aprotinin (33 µg/mL, Sigma-Aldrich A1153), inactivated horse serum (Gibco 26050), that was decreased gradually from 10% over 4% (for two days) to 0% (finally supplemented with triiodothyronine 0.5 ng/mL and hydrocortisone 50 ng/mL); relating to main manuscript methods section on "*Mechanical pro-hypertrophic intervention in EHTs*".

Media were pipetted into a 24-well plate with 1.5ml per well and equilibrated at least 30 minutes prior to EHT transfer. Experiments were conducted in the contractibility measurement system starting with the baseline measurement before preconditioning. The plates remained in the gas-flooded small incubator within the contractility measurement system until end of early reperfusion except for the described changes of the culture medium.

Method S3: Isolation of RNA and qPCR from EHTs

EHTs or human cardiac tissue samples (reviewed and approved by the regional ethics review board of the Medical Council of Hamburg, Germany, approval number 088/04) were homogenized with steel beads in a highly chaotropic isolation buffer. A phenol-chloroform based method (TRIzol, Ambion) was performed and total RNA was extracted from the aqueous phase utilizing isopropanol and silica columns (Lexogen Split RNA Extraction Kit). For reverse transcription of mRNA and the subsequent PCR with 40 cycles overall, the High Capacity cDNA Reverse Transcription Kit (Applied Biosystems) and HOT FIREPol EvaGreen qPCR Mix Plus (Solis BioDyne) were used according to manufacturers' instructions. PCR products were visualized on a 2%-agarose gel with Midori Green Advance DNA stain (NIPPON Genetics). The primer sequences for rat EHTs were as follows: Oprd1 (opioid receptor, delta 1) forward 5'- AGTTTTCGCTCCTCGCCAA-3' and reverse 5'- AGTGTACCGGACGATTCCAAA-3', GUSB (glucuronidase, beta) forward 5'- CCCGCATGTCCCCAAGACGG-3' and reverse 5'- CGGCACGGAAGCTCCACAGG-3'. The primer sequences for human EHTs were: OPRD1 (opioid receptor, delta 1) forward 5'- GCTCTTCGCCAACGCCTC-3' and reverse 5'- ATCTTAGTGTACCGGACGATGC-3', GUSB (glucuronidase, beta) forward 5'- ACGATTGCAGGGTTTCACCA-3' and reverse 5'- CACTCTCGTCGGTGACTGTT-3'.

Figure S1: Contractility measurement system

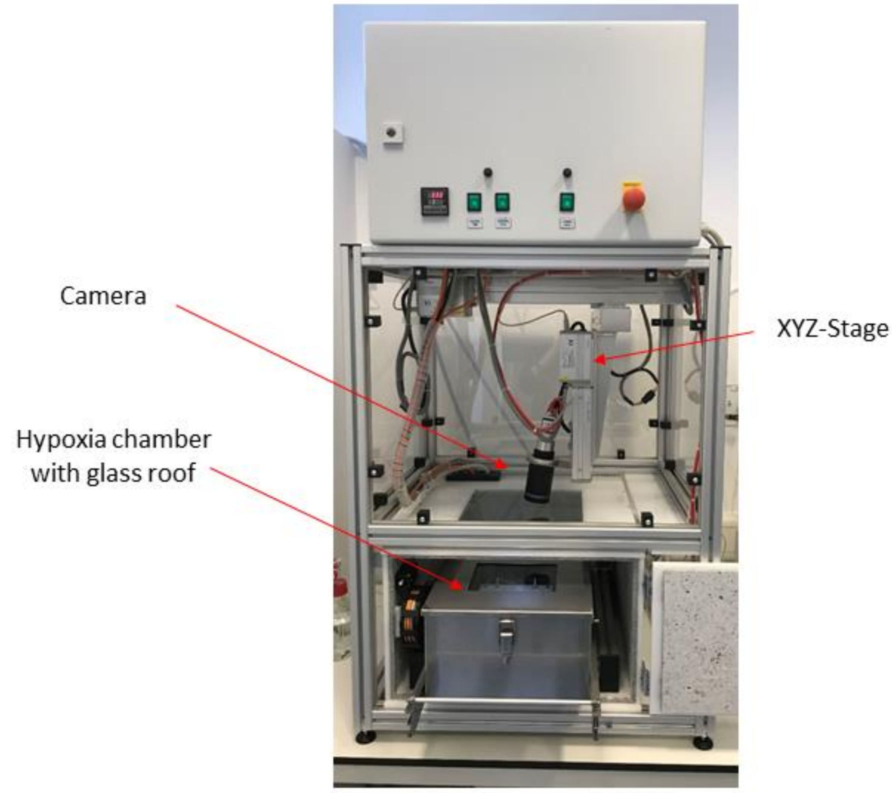

The automated contractility measurement system (EHT Technologies GmbH, Hamburg, Germany). One of its components is a small incubator (length ·width ·height = 30cm ·30cm ·17cm, volume 15.3 L) with a controllable gas inflow and a passive gas outflow. In the hypoxic periods the gas composition was 93% N_2 and 7% CO_2, in the baseline and reoxygenation periods as well as during other culture periods it was 53% N_2, 7% CO_2 and 40% O_2 (standard EHT culture environment). Probes in the small incubator report details on measured gas composition, humidity and temperature.

Table S1: Sources of compounds, reagents, cell lines and equipment used

Compound/reagent/ equipment/ cell line	Details	Manufacturer	City	State/Country
Culture medium: low glucose (1 g/L) DMEM	F0415	Sigma-Aldrich	Hamburg	Hamburg, Germany
Culture medium: minimal DMEM	Gibco A14430-01	ThermoFisher Scientific	Waltham	Massachusetts, USA
Inactivated horse serum	Gibco® 26050	ThermoFisher Scientific	Waltham	Massachusetts, USA
Penicillin/streptomycin	Gibco® 15140	ThermoFisher Scientific	Waltham	Massachusetts, USA
Insulin	I9278	Sigma-Aldrich	Hamburg	Hamburg, Germany
Aprotinin	A1153	Sigma-Aldrich	Hamburg	Hamburg, Germany
Matrigel	356235	BD Bioscience	Erembodegem	Belgium
Lactate (sodium lactate)	7455	Caesar and Loretz GmbH	Hilden	NRW, Germany
δ-opioid receptor agonist [D-Ala^2, D-Leu^5]-enkephalin (DADLE)	E7131	Sigma-Aldrich	Hamburg	Hamburg, Germany
Naloxon	Naloxon-ratiopharm	Ratiopharm	Ulm	Baden-Wuerttemberg, Germany
High-sensitivity rat cardiac Troponin I ELISA Kit	CTNI-2-HSP	Life Diagnostics Inc.	West Chester	PA, USA
Glucose-6-phosphate-dehydrogenase (G6PDH) detection kit	V-23111, *Vibrant*	Molecular Probes Europe BV	Leiden	The Netherlands

	Cytotoxicity Assay Kit			
ROS-GLO H_2O_2 Assay	G8820	Promega	Madison	Wisconsin, USA
RNA extraction kit (TRIzol, Ambion)	15596026	ThermoFisher Scientific	Waltham	Massachusetts, USA
RNA extraction kit	*Lexogen Split RNA Extraction Kit*	Lexogen	Vienna	Vienna, Austria
cDNA Reverse Transcription Kit	*High Capacity cDNA Reverse Transcription Kit*	Applied Biosystems (Thermo Fisher Scientific)	Waltham	Massachusetts, USA
PCR- DNA stain	Midori Green Advance DNA stain (MG04)	NIPPON Genetics Europe	Düren	NRW, Germany
qPCR mix	HOT FIREPol EvaGreen qPCR Mix Plus (08-24)	Solis BioDyne	Tartu	Estonia
Multimer-technology based UltraView Universal DAB Detection Kit	760-500	F. Hoffmann-La Roche Ltd.	Basel	Basel, Switzerland
Mouse anti myosin light chain 2, ventricular isoform (MLC-2V) monoclonal antibody	310111	Synaptic Systems GmbH	Göttingen	Lower Saxony, Germany
mouse anti-ANP monoclonal antibody	sc-80686	Santa Cruz Biotechnology	Dallas	Texas, USA
rabbit anti-active caspase-3 polyclonal antibody	AF835	R&D Systems, (Bio-Techne)	Minneapolis	Minnesota, USA

CC1-solution	950-124	Hoffmann-La Roche Ltd.	Basel	Switzerland
Wistar rats	Postnatal day 0-3	Charles River Laboratories Germany GmbH	Erkrath	NRW, Germany
Human cell line: hiPSC	UKEi003-C (information accessible at hpsreg.eu)	University medical center Hamburg-Eppendorf	Hamburg	Hamburg, Germany
Automated contractility measurement system	A0001	EHT Technologies GmbH	Hamburg	Hamburg, Germany
Microplate reader	TECAN Safire II	Tecan Trading	Männedorf	Zurich, Switzerland
Microplate luminometer	Centro LB 960	Berthold Technologies GmbH & Co. KG	Bad Wildbad	Baden-Württemberg, Germany
Microscope	Axioskop 2 microscope	Carl Zeiss Microscopy GmbH	Jena	Thuringia, Germany
Image processing program	ImageJ v1.53e	Wayne Rasband (retired from NIH)	Bethesda	MD, USA
Statistical analysis program	GraphPad Prism version 8.20	GraphPad Software	La Jolla	CA, USA

Video S1: Video of a spontaneously beating human EHT (see separate file)
